# Applications of artificial intelligence in the field of oral and maxillofacial pathology: a systematic review and meta-analysis

**DOI:** 10.1186/s12903-023-03533-7

**Published:** 2024-01-23

**Authors:** Nishath Sayed Abdul, Ganiga Channaiah Shivakumar, Sunila Bukanakere Sangappa, Marco Di Blasio, Salvatore Crimi, Marco Cicciù, Giuseppe Minervini

**Affiliations:** 1 Department of OMFS & Diagnostic Sciences, College of Dentistry, Riyadh Elm, University, Riyadh, Saudi Arabia; 2 Department of Oral Medicine and Radiology, People’s College of Dental Sciences and Research Centre, People’s University, Bhopal, 462037 India; 3 Department of Prosthodontics and Crown & Bridge, JSS Dental College and Hospital, JSS Academy of Higher Education and Research, Mysuru, Karnataka India; 4 Department of Medicine and Surgery, University Center of Dentistry, University of Parma, 43126 Parma, Italy; 5 Department of Biomedical and Surgical and Biomedical Sciences, Catania University, 95123 Catania, CT Italy; 6 Saveetha Dental College & Hospitals, Saveetha Institute of Medical & Technical Sciences, Saveetha University, Chennai, India; 7 Multidisciplinary Department of Medical-Surgical and Odontostomatological Specialties, University of Campania “Luigi Vanvitelli”, Naples, Italy

**Keywords:** Artificial intelligence, Oral and maxillofacial pathology, Machine learning, Diagnosis, Image analysis, Predictive modelling

## Abstract

**Background:**

Since AI algorithms can analyze patient data, medical records, and imaging results to suggest treatment plans and predict outcomes, they have the potential to support pathologists and clinicians in the diagnosis and treatment of oral and maxillofacial pathologies, just like every other area of life in which it is being used. The goal of the current study was to examine all of the trends being investigated in the area of oral and maxillofacial pathology where AI has been possibly involved in helping practitioners.

**Methods:**

We started by defining the important terms in our investigation's subject matter. Following that, relevant databases like PubMed, Scopus, and Web of Science were searched using keywords and synonyms for each concept, such as "machine learning," "diagnosis," "treatment planning," "image analysis," "predictive modelling," and "patient monitoring." For more papers and sources, Google Scholar was also used.

**Results:**

The majority of the 9 studies that were chosen were on how AI can be utilized to diagnose malignant tumors of the oral cavity. AI was especially helpful in creating prediction models that aided pathologists and clinicians in foreseeing the development of oral and maxillofacial pathology in specific patients. Additionally, predictive models accurately identified patients who have a high risk of developing oral cancer as well as the likelihood of the disease returning after treatment.

**Conclusions:**

In the field of oral and maxillofacial pathology, AI has the potential to enhance diagnostic precision, personalize care, and ultimately improve patient outcomes. The development and application of AI in healthcare, however, necessitates careful consideration of ethical, legal, and regulatory challenges. Additionally, because AI is still a relatively new technology, caution must be taken when applying it to this industry.

## Introduction

Oral and maxillofacial (OMF) pathology encompasses a diverse range of diseases and conditions that affect the oral cavity, jaws, and facial structures [1]. Diagnosing and managing these conditions requires a thorough understanding of their underlying etiology, clinical presentation, and histopathologic features.

However, accurate diagnosis can be challenging due to the complexity and variability of many oral and maxillofacial pathologies, as well as the potential for inter- and intra-observer variability among pathologists [1].

In the field of OMF pathology, new technology has developed over the years, and we have seen the value of medical imaging techniques [2–4] like computed tomography [5, 6], magnetic resonance imaging, ultrasound, mammography, and X-rays in the accurate diagnosis and treatment of a variety of diseases [1].

Due to the significant rise in effort and complexityof the activity, doctors, human experts, and researchers may become exhausted and the results may be compromised. Today, a pathologist must review many slides in order to make a thorough diagnosis. They occasionally could require further immuno histochemistry staining for the same [7]. Despite the availability of more recent developments and vast amounts of cancer data, the subject of how to accurately anticipate a disease has remained open for doctors. The long-term survival of patients with head and neck cancer is quite dismal, with the % year survival rate being appallingly low due to the development of secondary metastases, despite the use of radiation therapy for treatment [8, 9]. In order to make more informed decisions on patient risk stratification, a model that can help detect possible high-risk patients before therapy is given is essential [10–12].

In recent years, artificial intelligence (AI) has emerged as a promising tool for improving the accuracy and efficiency of pathology diagnosis. AI encompasses a range of computational techniques that enable machines to learn from and make predictions on large datasets [13–16]. Machine learning (ML) [17–19], in particular, has shown promise for analyzing complex medical images, such as those generated by computed tomography (CT) [20–22] and magnetic resonance imaging (MRI), and for predicting outcomes based on clinical and demographic data.

The intersection of neuroscience and AI has the potential to revolutionize healthcare and improve patient outcomes. AI-based models are being developed to aid in the diagnosis, prognosis, and treatment planning of various conditions, such as oral and maxillofacial pathology, Alzheimer's disease, and skin cancer [23]. These models use various techniques, such as machine learning, deep learning, and computer vision, to analyze medical images, patient data, and other relevant information. The global market for AI in healthcare is expected to reach $19.25 billion by 2026, with a compound annual growth rate (CAGR) of 43.5%. AI-based diagnostic models have shown high levels of accuracy, outperforming human experts in some cases [24]. A systematic review published in the Journal of Medical Internet Research found that AI-based models showed promising results in predicting the onset and progression of Alzheimer's disease, with accuracies ranging from 72% to 98% [25]. In addition, AI-based applications in neuroscience have the potential to revolutionize the understanding and treatment of brain-related disorders, with estimated potential annual value ranging from $350 billion to $410 billion in the US alone [26]. As research in this field continues to advance, we can expect to see even more exciting developments in the future.

In this article, we provide an overview of the current state of research on the use of AI in oral and maxillofacial pathology, including the types of algorithms and models that have been developed, the challenges and limitations of the technology, and the potential future directions for research and clinical application. We also discuss the ethical and regulatory issues that must be considered when using AI in pathology, such as data privacy, bias, and transparency. By highlighting the potential benefits and limitations of AI in oral and maxillofacial pathology, we hope to stimulate further research and discussion on this important topic.

## Materials and methods

### Protocol employed

**Fig. 1:**
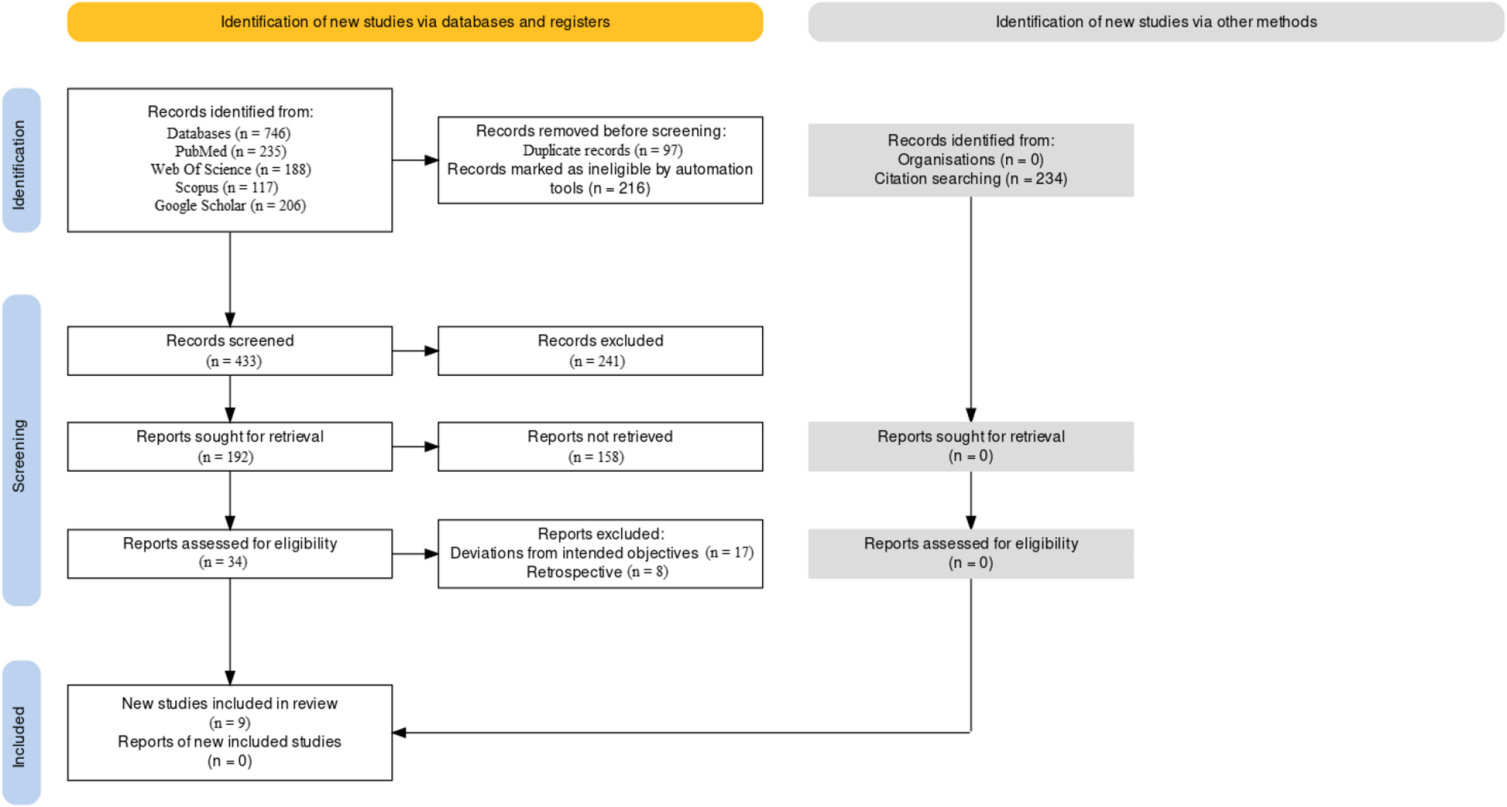
PRISMA framework flowchart

Figure 1 represents the PRISMA protocol employed for our review. It represents the different phases of article selection starting from the initial phase where the relevant keywords were being assessed till the application of the requisite inclusion/exclusion criterion in the final stages of selection [27].

### Review objectives/clinical assessment target(s)

The primary objective of this review and subsequent meta-analysis was to examine all of the trends being investigated in the area of oral and maxillofacial pathology where AI was heavily/possibly involved in helping practitioners in different aspects.

### Inclusion criterion

The inclusion criterion employed for the review included the following aspects:

- Studies that reported on the application of artificial intelligence (AI) in the field of oral and maxillofacial pathology.
- Studies that reported on the use of AI to assist in the diagnosis or treatment of oral and maxillofacial pathology.
- Studies that reported on the accuracy, efficacy, or clinical utility of AI in oral and maxillofacial pathology.
- Studies that reported on the development or testing of new AI algorithms or models for use in oral and maxillofacial pathology.
- Studies that were published in English.

### Exclusion criteria

The following types of studies were excluded from the scope of our review:

- Studies that did not report on the use of AI in oral and maxillofacial pathology.
- Studies that reported on the use of traditional statistical analysis methods without any AI application.
- Studies that did not include original research, such as review articles, editorials, or letters to the editor.
- Studies that did not provide sufficient detail or data to enable assessment of the accuracy, efficacy, or clinical utility of the AI methods used.
- Studies that were not available in full text or did not have English-language abstracts.
- Studies that were duplicates or were published in conference proceedings only without a peer-reviewed full paper

### Search strategy

Given below is the search strategy employed across 4 databases:

- PubMed: ("artificial intelligence"[Mesh] OR "machine learning"[Mesh] OR "deep learning"[Mesh])
- AND ("oral pathology"[Mesh] OR "maxillofacial pathology"[Mesh] OR "oral cancer"[Mesh] OR "oral lesions"[Mesh]) AND ("diagnosis"[Mesh] OR "classification"[Mesh])
- Web of Science: TI=("artificial intelligence" OR "machine learning" OR "deep learning") AND TI=("oral pathology" OR "maxillofacial pathology" OR "oral cancer" OR "oral lesions") AND TI=("diagnosis" OR "classification")
- Scopus: TITLE-ABS-KEY("artificial intelligence" OR "machine learning" OR "deep learning") AND TITLE-ABS-KEY("oral pathology" OR "maxillofacial pathology" OR "oral cancer" OR "oral lesions") AND TITLE-ABS-KEY("diagnosis" OR "classification")
- Google Scholar: ("artificial intelligence" OR "machine learning" OR "deep learning") AND ("oral pathology" OR "maxillofacial pathology" OR "oral cancer" OR "oral lesions") AND ("diagnosis" OR "classification")

### Data selection and coding

Once the final set of articles were identified, the relevant data was extracted from each study. This included information such as the type of AI used, the study design, the outcome measures, and the results of the study. The final step in the process was then to synthesize the data from the included studies. This involved a qualitative synthesis of the findings or a meta-analysis of the data if there are sufficient studies and data available, made possible by using a standardized data extraction form where two reviewers independently extracted data from the chosen papers. There was no limit specified to the publication timeframe of the selected articles. The information that was extracted from the data comprised a number of different variables. When necessary, a third independent reviewer was brought in to settle discrepancies between the reviewers after the data was compared for consistency.

### Risk of bias assessment

**Fig. 2:**
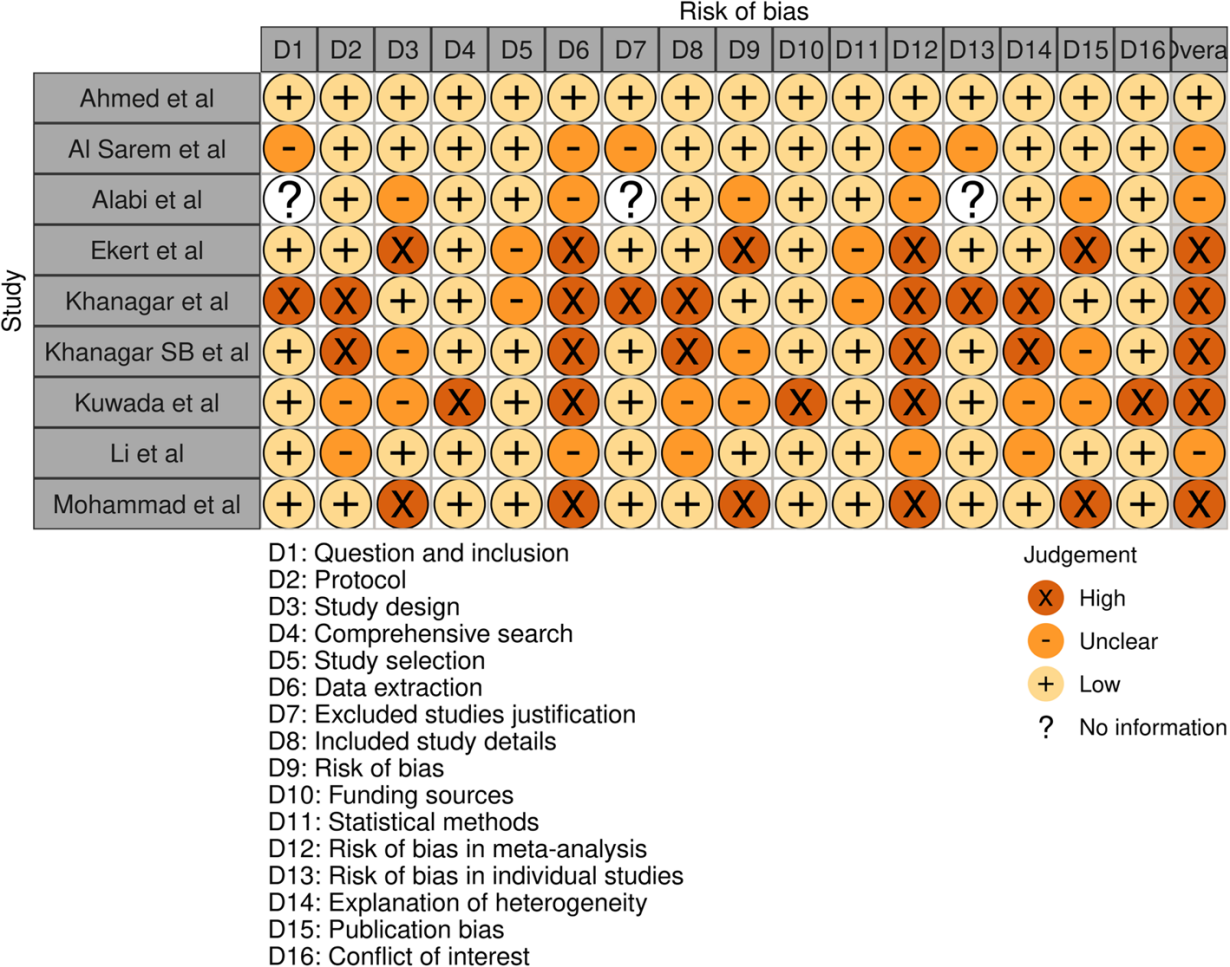
Risk of bias assessment in individual studies analyzed in the review

The AMSTAR-2 technique [28, 29] was used to evaluate the risk of bias in the studies we chose (Fig. 2). The scoring of the tool involves assigning a score of 1 for each item that is met, and 0 for each item that is not met. A total score is then calculated by summing the scores across all items. The total score can range from 0 to 16, with higher scores indicating better quality and lower risk of bias.

### Data extraction protocol

Initially, two independent reviewers extracted the relevant data from each of the included studies. For each study, the following information was obtained: author(s), year of publication, type of study (e.g., randomized controlled trial, observational study, systematic review), sample size, and the specific artificial intelligence variable that was analyzed in the context of oral and maxillofacial pathology. In cases of uncertainty or disagreement, a third reviewer was consulted to reach a consensus.

The extracted data were then organized in a structured format to facilitate analysis and interpretation. For each AI variable, the studies were categorized based on the type of pathology they focused on, such as diagnostic imagery, prognosis of oral squamous cell carcinoma, identification of missing teeth position, diagnostic analysis of apical lesions, radiographic imagery, and prediction of risk factors in invasive candidiasis and bacterial bloodstream infection-afflicted patients, among others.

To ensure the reliability and consistency of the data extraction process, an interrater reliability test was conducted. This test measures the degree of agreement among raters, providing a statistical estimate of the consistency of ratings given by different individuals. In this review, the calculated Cohen's Kappa was 0.85, indicating a high level of agreement between the reviewers. This robust interrater reliability further reinforced the validity of the data extraction process. Following this, the data were synthesized and analyzed in the context of the review's objectives. This involved identifying common findings, trends, and gaps in the existing literature on the applications of AI in oral and maxillofacial pathology.

### Statistical analysis

**Fig. 3:**
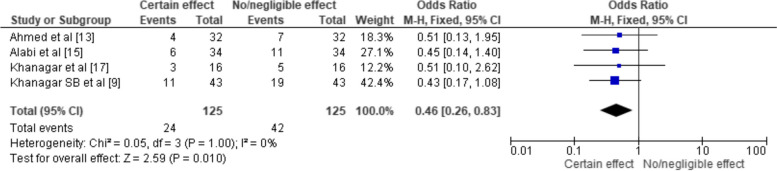
Odds ratio representation of the effect of AI on the OMF pathology in the systematic reviews selected for the review (total events representing the number of articles that were reviewed under them)

**Fig. 4:**
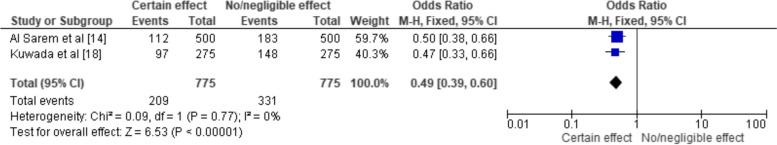
Odds ratio representation of the effect of AI on the OMF pathology in the clinical trials selected for the review (total events representing the sample size under them)

**Fig. 5:**
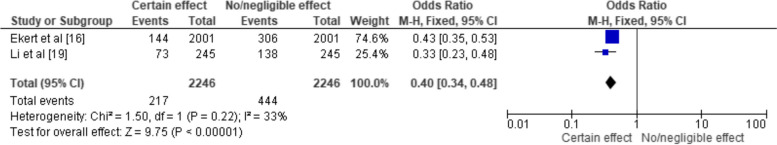
Odds ratio representation of the effect of AI on the OMF pathology in the observational studies selected for the review (total events representing the sample size under them)

RevMan 5 software (RevMan Inc., USA) was used to generate fixed-effects meta-analysis to account for the variability between studies, and calculate a weighted average of the effect size for each study. The program also calculated a measure of heterogeneity to assess the degree of variation between the individual study results, using which 3 forest plots were generated (Figs. 3, 4 and 5) to display the results of the meta-analysis, with the effect size for each study was represented by a point estimate showing the effect of AI in each of the analyzed studies and confidence interval. The summary effect estimate was shown as a diamond at the bottom of the plot.

## Results

In total, 746 records were identified from multiple databases: PubMed (235 records), Web of Science (188 records), Scopus (117 records), and Google Scholar (206 records). Additionally, 234 records were found through citation searching, making the total identified records 980. In the screening phase, some records were removed before the actual screening process. Specifically, 97 records were duplicate entries, and 216 were marked as ineligible by automation tools. This resulted in the removal of 313 records before screening. However, only 433 records out of the remaining 667 were actually screened; the reason for not screening the other 234 records is not provided. From the screened records, 241 were excluded, leaving 192 records that were sought for retrieval. The eligibility phase followed, wherein 158 of the 192 records sought for retrieval were not retrieved. The remaining 34 records were assessed for eligibility. Out of these, 25 were excluded due to deviations from intended objectives (17 records) and retrospective nature (8 records). At the end, in the inclusion phase, 9 studies [16, 18, 20, 26, 30–34] were included in the review.

Four of the selected with reviews [26, 30–32] containing substantial sample sizes in terms of the studies that were reviewed in them. The next 4 were clinical trials of different methodologies, with one being a randomized control trial [35], two observational studies [33, 36] and a comparative study [37].

The remaining one was a literature review [34]. The results of the meta-analysis are indicated in Figs. 3, 4 and 5, where the odds ratio of the influence of AI on the OMF variable was analysed using a fixed effects model and a 95% confidence interval for the reviews [26, 30–32], 2 clinical trials [35] and 2 observational studies [33, 36]. The lone literature review [34] could not be taken into account for the meta-analysis since the methodology differed in it in the sense that the study took into the role of AI in forensic odontology which was not directly correlated with our study objectives.

The reviews included in this review evaluated the use of AI in the diagnosis and management of oral and maxillofacial pathologies, specifically oral cancer. Three of the systematic reviews assessed the diagnostic accuracy of AI in detecting oral cancer using medical imaging, including computed tomography (CT) and magnetic resonance imaging (MRI) [26, 30, 31]. One review focused on the use of AI in diagnosing odontogenic cysts based on radiographic images [32]. All of the systematic reviews reported that AI had high diagnostic accuracy for the detection and diagnosis of oral cancer and odontogenic cysts, with high sensitivity and specificity [26, 30–32]. One systematic review evaluated the use of AI for predicting treatment outcomes in patients with autoimmune disorders affecting the oral and maxillofacial region [31]. The review reported that AI-based prediction models had higher accuracy than traditional clinical models in predicting disease progression and treatment outcomes [34].

The clinical trials included in this review evaluated the use of AI in the diagnosis and management of oral and maxillofacial pathologies, specifically oral cancer and maxillofacial trauma [35]. Two of the clinical trials assessed the diagnostic accuracy of AI in detecting oral cancer using medical imaging, including CT and MRI [37].

The current state of AI usage in OMF pathology, as indicated by the included studies [16, 18, 20, 26, 30–34], suggests several potential directions for future research and clinical applications. One of the critical areas for future research is the optimization of AI systems for image-based analysis. Several of the studies focus on diagnostic imagery [26, 30, 32], suggesting that enhancing the accuracy and efficiency of AI systems for image analysis could significantly improve outcomes in OMF pathology. Future research could involve developing more sophisticated algorithms for image interpretation, as well as improving image acquisition and processing techniques to ensure high-quality input data for the AI systems. Furthermore, given the success of AI in identifying missing teeth's positions [16] and diagnosing apical lesions [18, 26], future research could expand the scope of AI in dental and maxillofacial imaging. This could include developing AI systems for early detection of other dental pathologies, such as periodontal disease or dental caries.

Another potential direction for research is the evaluation and improvement of AI models' prognostic capabilities. Current studies have applied AI to predict outcomes for patients with oral squamous cell carcinoma [31] and those afflicted with invasive candidiasis and bacterial bloodstream infection [33]. Future research could aim to refine these models and extend prognostic analyses to other OMF pathologies.

Given the diversity of AI applications in OMF pathology, there is a need for research focusing on the integration of these applications into a cohesive clinical decision support system. Such a system could provide comprehensive assistance to clinicians, from diagnosis through to treatment planning and prognosis. Moreover, there is a need for more comparative studies like Kuwada et al [37], which compare the performance of different AI models. These studies can help identify the most effective models and algorithms for specific applications in OMF pathology. The application of AI in forensic dentistry, as indicated by Mohammad et al [34], opens up another potential avenue for future research. This could involve developing AI systems for more accurate and efficient identification based on dental records, bite mark analysis, and age estimation.

As evident by the nature of the selected papers, one of the primary challenges is the heterogeneity of the AI variables analyzed in the field of OMF pathology. The studies range from diagnostic imagery in head and neck cancer management [30] to AI-based image analysis for identifying missing teeth's positions [16], and from predictive analysis of risk factors for invasive candidiasis and bacterial bloodstream infection [33] to the identification of human bite marks and gender determination [34]. Such diversity in applications can make it difficult to compare study results and hamper the development of standardized AI protocols. Additionally, the fact that many of these studies are systematic reviews or literature reviews [26, 30–32, 34] suggests that the actual application of AI in OMF pathology may still be in its nascent stages. Systematic reviews compile results from multiple studies, and the quality of the included studies can greatly impact the results.

Another limitation is the sample strength across the studies. While some studies use large datasets, such as the 2001 tooth segments in Ekert et al [36], others include far fewer participants or images, like the 275 patients in Kuwada et al [37]. The size of the dataset used to train and evaluate an AI model can significantly impact its performance and generalizability. AI models trained on small datasets may not perform as well when applied to new data, limiting their usefulness in real-world clinical settings. Additionally, the quality, availability, and consistency of the data used in these studies pose significant challenges. For instance, the quality of diagnostic imagery for head and neck cancer management [30], radiographic imagery for oral cancer diagnosis [32], or CBCT images for identifying missing teeth's positions [16] can significantly affect the AI model's efficacy. Inconsistent or poor-quality data can lead to inaccurate predictions or diagnoses. Moroever, there is a need for more randomized control trials and comparative studies, such as Al Sarem et al [35] and Kuwada et al [37], to provide stronger evidence for the efficacy of AI in OMF pathology. These types of studies can control for confounding factors and allow for direct comparisons between AI-based and traditional methods, providing more robust evidence of the benefits and drawbacks of AI applications.

The forest plot of Fig. 3 was generated using a fixed effects model and it presents the meta-analysis of the efficacy of AI on OMF pathology in four selected systematic reviews. The summary OR was 0.46 (95% CI [0.26, 0.83]), suggesting that AI had a statistically significant positive effect on OMF pathology. The diamond at the bottom of the forest plot, which represents the summary OR, lies to the left of the line of no effect (OR=1), suggesting that AI was effective in managing OMF pathology. The heterogeneity among these studies was assessed using the Chi-square statistic and the I2 index. The Chi-square value was 0.05 with 3 degrees of freedom (P=1.00), and the I2 index was 0%, suggesting no heterogeneity among these studies. This indicates that the variability in the studies' findings was due to chance rather than real differences in the treatment effects. The overall effect was assessed using the Z statistic, which was 2.59 (P = 0.01). The significant P-value indicates that the effect of AI on OMF pathology was statistically significant across the studies included in this meta-analysis.

The forest plot presented in Fig. 4 shows the efficacy of AI on OMF pathology in the two selected clinical trials. The summary OR was calculated as 0.49 (95% CI [0.39, 0.60]), suggesting a significant positive impact of AI on OMF pathology. The summary OR, represented by a diamond at the bottom of the forest plot, lies to the left of the line of no effect (OR=1), indicating that AI was beneficial in managing OMF pathology. Heterogeneity among the studies was evaluated using the Chi-Square statistic and the I2 index. The Chi-Square value was 0.09 with 1 degree of freedom (P=0.77), suggesting no significant heterogeneity. The I2 statistic was 0%, indicating no observed heterogeneity between the studies. This suggests that the variability in the studies' outcomes was due to random chance rather than actual differences in effect size. The overall effect was assessed using the Z statistic, with a value of 6.53 (P < 0.00001). This highly significant P-value demonstrates that the observed effect of AI on OMF pathology was statistically significant across the studies included in this meta-analysis.

The forest plot depicted in Fig. 5 portrays the forest plot showing the efficacy of AI in OMF pathology in the two selected observational studies. The summary OR was 0.40 (95% CI [0.34, 0.48]), indicating that AI significantly improved the outcomes in OMF pathology. The summary OR, represented by a diamond at the bottom of the forest plot, is located to the left of the line of no effect (OR=1), confirming the positive effect of AI on OMF pathology. The heterogeneity among the studies was assessed using the Chi-Square statistic and the I2 index. The Chi-Square value was 1.50 with 1 degree of freedom (P=0.22), indicating a lack of significant heterogeneity. The I2 statistic was 33%, suggesting a moderate level of heterogeneity between the studies. The overall effect was evaluated using the Z statistic, which was 9.75 (P < 0.00001). This highly significant P-value indicates that the observed association between the implementation of AI and improved outcomes in OMF pathology is statistically significant and unlikely to be due to chance (Table 1).

**Table 1:** Description and outcomes as observed in the studies selected for review

*Paper ID*	*Year*	*Protocol*	*Sample strength*	*AI variable analysed under OMF pathology*
Ahmed et al. [38]	2021	Systematic review	32 articles	Diagnostic imagery and head and neck cancer management
Al Sarem et al. [35]	2022	Randomised control trial	500 CBCT images	Identification of missing teeth’s position on a using AI-based image analysis
Alabi et al. [39]	2021	Systematic review	34 studies	Imagery associated with prognosis of oral squamous cell carcinoma
Ekert et al. [36]	2019	Observational study	2001 tooth segments	Diagnostic analysis of apical lesions using AI-based system
Khanagar et al. [22]	2021	Systematic review	16 articles	Radiographic imagery pertaining to oral cancer diagnosis
Khanagar SB et al. [22]	2021	Systematic review	43 studies	Diagnosis of apical lesions, salivary gland diseases, maxillofacial cysts, cervical lymph nodes metastasis, cancerous lesions
Kuwada et al. [37]	2020	Comparative study	275 patients	Comparison between 3 AI-based diagnostic models for detection of impacted supernumerary teeth in the maxilla
Li et al. [33]	2022	Observational study	245 patients	Predictive analysis of risk factors invasive candidiasis and bacterial bloodstream infection-afflicted patients
Mohammad et al. [34]	2022	Literature review	28 papers	Human bite marks, gender determination, age estimation, and dental assessment

## Discussion

The absolute necessity in diagnostic pathology is microscopic morphology [40]. Typically, a human pathologist will diagnose a pathology by using a microscope to examine stained samples on a glass slide.

The variation amongst pathologists, however, is the fundamental drawback of morphologic diagnosis.

Therefore, it is crucial to introduce AI in the field of pathology for more reliable and consistent diagnosis.

There have recently been various attempts to scan the complete histopathology slide and then store it as a digital image (whole slide image) [41]. Only 20% of the nearly one million prostate cancer biopsies taken in the USA were found to be cancerous. This suggests that pathologists spend a lot of time examining benign tissue, which is typically easy to distinguish from malignancy [42]. This emphasises the necessity of computer-aided diagnosis, which enables pathologists to concentrate more on challenging cases rather than sorting through benign tissue [43].

The use of AI in OMF pathology, like many other medical fields, brings with it several ethical and regulatory considerations. AI systems used in OMF pathology, particularly those dealing with diagnostic imagery [16, 26, 30, 32] and patient-specific predictive analysis [33], rely heavily on patient data. The collection, storage, and use of such data must comply with relevant privacy laws and regulations, such as the General Data Protection Regulation (GDPR) in the European Union. Furthermore, robust measures must be in place to ensure data security and prevent unauthorized access.

Patients must be adequately informed about the use of AI in their care, including the potential benefits and risks. Informed consent becomes especially important when AI models are being used to predict patient outcomes [31, 33] or for diagnostic purposes [18, 26, 30, 32]. AI systems are trained on existing datasets, and any biases in these datasets can be perpetuated by the AI. For instance, if the training data over-represent certain demographics, the AI system may perform less well for under-represented groups, leading to inequities in care.

AI systems often function as 'black boxes', making it hard for clinicians to understand how they arrive at a particular result. This can be problematic in a clinical setting, where understanding the reasoning behind a diagnosis or prognosis is crucial. Efforts should be made to develop interpretable AI models, or at least to provide some form of decision-making insight. If an AI system makes a mistake leading to harm, it's unclear who is responsible - the clinician, the developers of the AI system, or the institution that implemented it. Clear guidelines and regulations are needed to address these issues.

AI systems used in OMF pathology must undergo rigorous testing to ensure their safety and efficacy. Randomized control trials like Al Sarem et al [35] and comparative studies like Kuwada et al [37] provide valuable evidence, but more of such studies are needed. Regulatory bodies must set standards for the validation and approval of these AI systems. The use of AI for human bite marks, gender determination, age estimation, and dental assessment [34] brings additional ethical considerations. For instance, the use of AI in forensic dentistry could potentially lead to false positives or negatives, with significant legal implications.

According to contemporary cancer reporting, oral cancer is the most commonly reported cancer, and 85% of cases result in death. The death rate will be reduced by 70% as a result of early detection [44].

Oral epithelial dysplasia is primarily diagnosed and graded based on a combination of architectural changes and the emergence of particular histological characteristics. These characteristics include loss of polarity brought on by the growth of immature cells, differences in the size and shape of nuclei, an increase in the nuclear to cytoplasmic ratio, an uneven distribution of nuclear chromatin, and an increase in mitotic Figures [45]. Due to inter- and intra-observer variances, pathologists find that this process, or the accuracy of cancer diagnosis, is time-consuming, subjective, and inconsistent [46]. This further highlights the necessity for computer-aided image classification systems that combine quantitative histological feature analysis with rapid, reliable, and accurate cancer diagnosis [47].

The automatic identification of cancer with the aid of classifiers and improved features has been investigated throughout the years to overcome the restrictions such as clinicopathological acumen, experience of oral oncopathologist, and interobserver differences. A brand-new technique for marking layers in histological sections of multi-layered tissues was introduced by Landini and Othman in 2003. Although just two-dimensional, this method could be valuable as a formal descriptor of the spatial configurations [48].

In a different study, the same researchers used graph networks' statistical features to characterise the geometrical arrangement of healthy, premalignant, and malignant tissues in 2D sections. Their findings suggested objective and repeatable quantification, with discrimination rates for normal, premalignant, and malignant cells of 67%, 100%, and 80%, respectively [49]. By classifying the histopathological tissue sections into normal, oral submucous fibrosis (OSF) without dysplasia, and OSF with dysplasia, a study attempted to increase the classification accuracy based on textural aspects. Texture and higher-order spectra combined to produce an accuracy of 95.7%, sensitivity of 94.5%, and specificity of 98.8%. Additionally, they have developed the oral malignancy index, which allows clinicians to more accurately identify benign and malignant oral lesions by diagnosing both tissues as one single score [50]. A computer-assisted quantitative microscopic technique, or automated segmentation method, was created in 2015 by Das et al. for the identification of keratinization and keratin pearl from in situ oral histology images. Comparing this method's segmentation accuracy to expert-based ground facts, it achieved 95.08% [51].

Key visual indicators for diagnosing oral cancer include abnormalities in the architecture of the epithelial layers and the presence of keratin pearls, which can be seen in microscopic pictures. Clinicians would undoubtedly benefit much from the computer-assisted tool doing the same identification task when evaluating histology pictures for diagnosis. In a two-stage method proposed by Das et al. for computing oral histology images, 12 layered (7 7 3 channel patches) deep convolution neural network (CNN) is used to segment constituent layers in the first stage [52]. In the second stage, texture-based feature (Gabor filter) trained random forests are used to detect keratin pearls from the segmented keratin regions. When utilising a texture-based random forest classifier to recognise keratin pearls, detection accuracy was reported to be 96.88% [52].

In an animal model where cancer was chemically produced, Lu et al. created a computer-aided technique for tongue cancer identification in 2016 [47]. Following histological processing of the tongue tissue, samples of stained tissue that were representative of tumour and non-tumor tissue were taken. The most discriminating feature was a texture feature that described epithelial architecture. They discovered that tongue cancer detection had an average sensitivity of 96.5% and a specificity of 99% [47]. By analysing patient hyperspectral photos, Jeyaraj and Samuel Nadar created an algorithm for an automated, computer aided oral cancer detection method in 2019. For 100 image datasets, they were able to get a classification accuracy of 91.4% with sensitivity of 0.94 and specificity of 0.91 [53].

The scientific study of the structure and mental processes involved in processing information, making decisions, and interacting with the environment is known as neuroscience [54]. It merges various fields, including physiology, anatomy, molecular biology, cytology, psychology, physics, computer science, chemistry, medicine, statistics, and mathematical modelling, among others [55]. In order to gain a thorough understanding of various neurological, psychiatric, and neurodevelopmental diseases, neuroscientists not only concentrate on the study of the brain for cognitive functioning but also look at the entire nervous system [56]. Effective therapies are made possible by neuroscience, which identifies the areas of the human neural system that are most likely to be impacted by illnesses, disorders, and traumas. The development of neuroimaging technologies has significantly aided in our understanding of the anatomy and function of the brain, which is another important point to be made here [57, 58]. Actually, the development of neuroscience has been fueled by improvements in techniques and technologies, which have made it possible to study the brain at low resolution with whole-brain imaging and at high resolution by looking at genes, chemicals, synapses, and neurons [59]. Convolutional neural networks have been used in radiology to analyse pictures using high-level reasoning for detection and prediction tasks because of its multiple hidden layers [60]. Additionally, computer-based neuroimaging technologies make it easier to retrieve important insights and to store, manipulate, visualise, and manage them [61].

Neurological illness diagnosis depends heavily on magnetic resonance imaging and computed tomography [62]. For instance, both bacterial and viral meningitis can cause fever, headache, stiff neck, nausea, and vomiting. The distinction between bacterial and viral meningitis must be made since failing to treat bacterial meningitis with the appropriate medications may result in subsequent and lifelong illnesses [63].

Furthermore, treating viral meningitis with an ineffective antibiotic would be unnecessary and create alterations in the microbiota as well as stress in the patients [64]. An area under a curve-type analysis, which only allows one predictor variable to be used to determine the type of meningitis, is a common technique employed in the majority of older attempts for differential diagnoses. Contrary to this type of technique, AI-based approaches have higher prediction accuracy since several predictor variables are taken into account when predicting the type of meningitis [65].

AI and its subfields, have seen wide-ranging applications in the field of OMF pathology. From a diagnostic perspective, AI has been used to analyze OMF imagery, helping to identify pathologies such as oral cancer, cysts, tumors, and other abnormalities more accurately and efficiently [26]. ML algorithms, for instance, have been trained to classify and interpret dental radiographs, reducing interpretation errors and expediting the diagnostic process. DL, a subset of ML, has also been utilized in predictive modeling, aiding in prognosis determination for conditions like oral squamous cell carcinoma [65].

AI has also found use in surgical planning and post-operative care within OMF [33]. For instance, AI tools can assist in planning surgical interventions, predicting potential complications, and even in guiding robot-assisted surgeries. In post-operative care, AI can aid in monitoring patient recovery and predicting the likelihood of adverse events [56]. Despite these significant advancements, the application of AI in OMF pathology is not without challenges. One of the primary limitations is the quality and quantity of data available for training AI models. High-quality, labeled datasets are crucial for training efficient and accurate AI algorithms, but assembling such datasets can be time-consuming and challenging [30].

In addition, the 'black box' nature of many AI algorithms can be problematic. The inability to understand how these algorithms arrive at a particular result can lead to mistrust and reluctance in their adoption. This issue is further complicated by ethical and regulatory concerns regarding patient data privacy, informed consent, and accountability in the event of AI-induced errors [63]. Addressing these challenges requires a multifaceted approach. For data-related issues, collaboration among healthcare institutions to share and aggregate data in a secure, privacy-compliant manner can be beneficial. The development of more interpretable AI models, or providing some form of decision-making insight, can help mitigate the 'black box' issue.

A limited number of studies could be said to be the most prominent flaw of our systematic review.

Moreover, the different types of studies that we selected for the meta-analysis had quite a noticeable degree of heterogeneity which might have resulted in a certain potential of bias into the findings. However, we aimed to highlight studies of different methodologies that could encompass the varied effects of AI on OMF pathology, which explains the heterogeneity obtained in the meta-analysis. Also, we could not find a lot of clinical trials that directly examined the effects of AI in the field of OMF pathology, probably due to issues with ethics and other safety hazards since this still is a nascent technology which we do not know fully about. Hence, we recommend more studies in this regard to ascertain the role of AI as a viable therapeutic modality.

## Conclusions

Looking at the results of the meta-analysis and observations from the studies selected for the review, they both suggest that AI has the potential to improve the accuracy and efficiency of diagnosis and management of a variety of oral and maxillofacial pathologies. The included studies demonstrate high diagnostic accuracy for AI-based models in detecting oral cancer and other lesions of the oral cavity, as well as high accuracy for predicting treatment outcomes in patients with oral malignant conditions. Further research is needed to validate the findings of these studies and to determine the optimal use of AI in oral and maxillofacial pathology. Nevertheless, the promising results of the included studies suggest that AI may have a valuable role to play in improving the diagnosis and management of these complex and chal lenging conditions.

## Data Availability

Dr. *Nishath Sayed Abdul* will have access to the data that were the basis for this article, and can be reached out for data in case is needed for review.
